# Openness and trust in data-intensive science: the case of biocuration

**DOI:** 10.1007/s11019-020-09960-5

**Published:** 2020-06-10

**Authors:** Ane Møller Gabrielsen

**Affiliations:** NTNU University Library, Trondheim, Norway

**Keywords:** Biocuration, Open science, Data-intensive science, Trust

## Abstract

Data-intensive science comes with increased risks concerning quality and reliability of data, and while trust in science has traditionally been framed as a matter of scientists being expected to adhere to certain technical and moral norms for behaviour, emerging discourses of open science present openness and transparency as substitutes for established trust mechanisms. By ensuring access to all available information, quality becomes a matter of informed judgement by the users, and trust no longer seems necessary. This strategy does not, however, take into consideration the networks of professionals already enabling data-intensive science by providing high-quality data. In the life sciences, biological data- and knowledge bases managed by expert biocurators have become crucial for data-intensive research. In this paper, I will use the case of biocurators to argue that openness and transparency will not diminish the need for trust in data-intensive science. On the contrary, data-intensive science requires a reconfiguration of existing trust mechanisms in order to include those who take care of and manage scientific data after its production.

## Introduction

In 2010, the European Commision’s High-Level Group on Scientific Data stated that “[w]e are on the verge of a great new leap in scientific capability, fueled by data” (European Commission 2010, p. 9). Ten years later, terms like “data-driven science”, “data-intensive science”, and “e-science” are gaining traction within what is sometimes described as a paradigmatic shift in the conditions for knowledge production. One of the key factors of this shift is the combination and integration of the enormous amounts of data being generated. Another factor is the belief that the data will speak for itself through computational analyses without the need for prior theories, models or hypotheses (Kitchin 2014). Although the claims of a new and “theory-free” science generating knowledge from Big Data have been questioned by both scientists and philosophers of science (e.g., Kitchin 2014; Leonelli 2016), there are still enough changes taking place to warrant the label “data intensive” science. New technologies for data generation, dissemination and interpretation are reshaping scientific knowledge infrastructures and data has gained status as a valuable scientific output in its own right (Leonelli 2016).

According to philosopher of science Sabina Leonelli, data-intensive science requires more *trust* as the distributed nature of data dissemination systems limits the ability of individuals to understand the systems as a whole (Leonelli 2016). However, scientific trust is already challenged (e.g.,Barber 1987; Sarewitz 2016). As Richard Horton, editor-in-chief of the scientific journal *The Lancet*, argues, something seems to be wrong with the way science is currently conducted:

The case against science is straightforward: much of the scientific literature, perhaps half, may simply be untrue. Afflicted by studies with small sample sizes, tiny effects, invalid exploratory analyses, and flagrant conflicts of interest, together with an obsession for pursuing fashionable trends of dubious importance, science has taken a turn towards darkness (Horton 2015, p. 1380).

In other words, it seems as if science cannot be completely trusted. Or, rather, that *scientists* do not necessarily adhere to the technical and moral norms that are a prerequisite for believable knowledge claims (Barber 1987).

The risks are even higher with data-intensive science. In order to fully utilize the enormous amounts of data, science policies call for data sharing, but sharing of large quantities of information through digital technologies comes with its own challenges. When the amounts of available information increase, so does the risk of inaccuracies, flaws and even fraud as researchers “have no real way of knowing for sure if, on the other end of the line, they will find man or machine, collaborator or competitor, reliable partner or con-artist, careful archivist or data slob” (European Commission 2010, p. 17).

In current European science policies, *openness* is presented as a means to more credible and reliable science (European Commission 2016). Through openness and transparency, data will be available for validation, scientific integrity will be encouraged and public trust in science will eventually be restored. In other words, in addition to enabling data-intensive science by making data and information accessible, openness is supposed to mend the situation described by Horton above. However, implicit in this claim is also the premise that there is no longer really any need to trust anyone as everything will be transparent and accessible for scrutiny.

The life sciences are often perceived to be in the forefront of data-intensive science (Nielsen 2011; Leonelli 2016). Biological databases have become central for biological research (Kersey and Apweiler 2006; Leonelli 2013) and these databases are usually populated and managed by expert *biocurators*. In this paper, I will use the example of biocurators to argue that data-intensive science requires not a dismissal, but a reconfiguration of scientific trust. As trust is a complex phenomenon, I will start by giving a short overview of some important approaches to scientific trust followed by an equally short analysis of how trust is framed in European visions and policies concerning data-intensive science.

## Trust in science

Although several disciplines have taken different angles at conceptualizing trust without reaching any consensus (Misztal 1996), a common understanding is trust as a certain kind of positive assumptions or expectations about others and their actions (Luhmann 2000; Barber 1987; Hendriks et al. 2016). Trust, understood in this manner, is therefore a matter of interpersonal trust; it concerns relations between people. Although trust is considered essential for almost any kind of social cooperation and interaction, it is often viewed as redundant in science. Robert Merton’s famous account of the scientific ethos includes organized skepticism (Merton 1942) and scientific knowledge is often assumed to be evidence-based and emerging only after rounds of systemic distrust where all inaccuracies and false claims have been washed away and eliminated. Trust, on the other hand, involves a certain level of vulnerability and risk of disappointment; it does not offer any sort of guarantee that people will live up to the expectations. Quite the contrary, as philosopher Onora O’Neill points out; when outcomes are guaranteed, trust is redundant (O'Neill 2002).

However, as philosopher John Hardwig argues in “The Role of Trust in Knowledge” (Hardwig 1991), trust is not only necessary in science; it might be even more epistemically important than empirical data and logical arguments. As the traditional mechanisms for ensuring reliability—peer review and replication—have proved to be insufficient in order to detect fraud and poor-quality research, Hardwig argues that researchers are left with no option than to trust fellow scientists in order to be able to produce new knowledge (Hardwig 1991). Without trust, scientists would be left in in “experimenter’s regress”, constantly forced to replicate experiments in order to verify them (Bijker et al. 2016) and scientific knowledge therefore rests on a foundation built on the trustworthiness of members of epistemic communities (Hardwig 1991).

In “Trust in Science” (1987), sociologist Bernhard Barber argues that trust in science is not much different from trust elsewhere in society and distinguishes between two relevant types of trust: One concerning the expectation that someone will competently perform an assigned task, the other concerning the expectation that someone will observe and fulfill their fiduciary obligations and responsibilities and put them above their own interests (Barber 1987). In other words, scientists must be expected to adhere to certain technical and moral norms and criteria in order to produce believable knowledge claims. Furthermore, according to Barber, trust also concerns those who assess and evaluate scientific knowledge claims; they must be believed to be both competent and responsible in order to be trustworthy (ibid.). Trust in science thus depends on a chain of assumed trustworthy elements starting with the original knowledge producers and ending with review by assumed trustworthy peers before publication. Although obvious failure or violation of the norms could be met by formal sanctions like withdrawal of funding or even legal action, Barber notes that the scientific community prefers to rely on social processes, like informal sanctioning by colleagues, in order to maintain trust (Barber 1987).

## Data-intensive science: openness replacing trust?

According to Barber, there has been no substantial change in the formal and informal maintenance of scientific trust relations since the academies rose to forefront of scientific power in the eighteenth century (Barber 1987). Science, however, has changed since Barber made his observations. Data is now considered a valuable scientific output beyond its original cause for production, and according to some, we are at the verge of a fourth scientific paradigm of data-intensive science enabled by new digital technologies for data production, sharing and analysis (Hey et al. 2009).

In order to utilize the enormous amounts of data, calls for openness and sharing are becoming increasingly common, both in science policies and in scholarly communities. Open Data is considered to be a fundamental element of Open Science, which again is presented as “a systemic change to the way science and research have been carried out for the last fifty years” (European Commission 2016). It is important to note that in addition to increasing efficiency and collaboration by making data and information readily available, Open Science is also expected to enhance transparency and accountability (Levin and Leonelli 2017) and therefore also the potential trustworthiness of research. By having access to all the underlying data, everyone will be able to check and validate results, and by having access to sufficient *metadata*, i.e., additional information about the data and how it was produced, potential reusers will be able to make informed judgements about the quality and reliability of the data itself (European Commission 2018a).

In Europe, the quest for Open Science is manifesting through The European Open Science Cloud, “a trusted, open environment for the scientific community for storing, sharing and re-using scientific data and results” (European Commission 2018a, p. 16). A key concept for the envisioned cloud is *trusted repositories*; certified digital repositories that fulfill certain requirements (European Commission 2018a). In addition to providing full transparency of their services, trusted repositories should also provide *FAIR* compliancy in order to provide the best conditions for quality control and subsequent reuse (European Commission 2018b). The FAIR-principles (Findable, Accessible, Interoperable and Reusable) advocate the consistent use of identifiers, standards and metadata in machine-readable formats (Wilkinson et al. 2016) that in turn will enable users to assess quality and reliability. As stated in the European Commission report *Turning FAIR into Reality*:

The rich metadata and provenance information required to achieve Reusability should include details that address data assessability. It is important to provide information that allows potential (re)users to judge the accuracy, reliability and quality of the data, and to determine whether these data meet their needs (European Commission 2018b, p. 22).

According to Grand et al., Open Science has the potential to become a new “trust technology” benefiting both the scientific community and the public by complementing or even replacing existing trust systems (Grand et al. 2012). However, as O’Neill (2002) points out, openness does not necessarily increase trust. As already mentioned, trust involves positive expectations about the actions of others that may or may not be accurate and involves quite a bit of risk that these expectations may be wrong. Openness aims to limit this risk, thereby making trust in other peoples’ actions and intentions redundant. In the Open Science-scenario, there is no real need to trust anyone as everything will be available for checking and validation, and in this sense, Open Science is therefore rather a “trust-no-one-technology”.

## Biocuration: enabling data-intensive biology

Due to new technologies for generation of biological data, the life sciences are said to be well on their way to becoming data-driven, or data-intensive. As an article in *Nature* put it: “Biologists are joining the big-data club” (Marx 2013, p. 255). According to Leonelli, this does not necessarily mean that the life sciences are entering a data-driven paradigm where knowledge is extracted from large amounts of data without previous hypotheses, but rather that they are turning towards a *data-centric* approach to science “within which efforts to mobilize, integrate, and visualize data are valued as contributions to discovery in their own right and not as a mere by-product of efforts to create and test scientific theories” (Leonelli 2016, p. 1).

Although biological databases have existed since the 1960s, the “data deluge” of the life sciences started in the 1990s when the Human Genome Project was officially launched and gave rise to a massive amount of publicly available sequence data. In a short piece in *Nature* 1991, molecular biologist Walter Gilbert at Harvard University claimed that biology was facing a paradigm shift: The soon-to-be-realized knowledge of all the genes would guide all future biological research, and the vessel for the shift would be the biological databases (Gilbert 1991). Gilbert saw the potential of the “flood of knowledge” that would soon be available, but also understood that it came with challenges: “The next tenfold increase in the amount of information in the databases will divide the world into haves and have-nots, unless each of us connects to that information and learns how to sift through it for the parts we need” (Gilbert 1991, p. 99).

This sifting is currently the responsibility of biocurators; “professional scientists who collect, annotate, and validate information that is housed within biological databases” (Research Information Network 2010). Although their number is rather small, the importance of biocurator efforts is invaluable due to the impact curated databases have had as almost every form of life science research involves the use of biological databases in one way or another, whether it is for looking up information on a certain gene or molecular interaction, fuelling analyses of large amounts of sequence data, or enabling computational models simulating the processes of biological systems.

According to Leonelli, the core task of biocuration lies in the *decontextualization* and *recontextualization* of data—the processes where data is detached from their original context through standardized terms and then provided with metadata, “reliability labels”, which allow the user to understand and evaluate how the data was produced (Leonelli 2016). As already mentioned, metadata is considered to be crucial for the possibility to assess the reliability of the data as it provides users with information to make informed judgements (e.g., Leonelli 2016; Marchionini et al 2012). In addition, biocurators might provide data with their own confidence rankings, giving the users an instant sense of the reliability (Leonelli 2016). In other words, biocurators provide services that allow users to get access to quality-checked information in addition to providing them with the tools for making their own evaluations when necessary.

Although important enough, biocuration involves more than providing metadata and confidence rankings in the correct formats. Beyond the issue of how to deal with the “data deluge” lies the even more complex question of how to incorporate new research output with what is already known. As Attwood et al. comment, new information is useless unless it is “stored and organized in ways that allow us to access it, to analyse it, to annotate it and to relate it to other information” (Attwood et al 2009, p. 318) and in addition to serving as archives for research data output, several biological databases are so-called added-value databases, or *knowledgebases*, which “build on archival resources by providing expert curation, annotation, reanalysis, and integration of archived experimental data” (Cook et al 2019, p. D17). A large part of biocuration work therefore consists of reading papers and translating relevant information into computer-readable formats, thereby enabling computational integration with new research output (Howe et al 2008).

Data *quality* is not included in the FAIR principles and “trusted repositories”-approach of Open Science as it is considered too difficult to standardize (European Commission 2018a). The Open Science Cloud will therefore “need to operate under the principle of let the buyer beware (caveat emptor) (European Commission 2018b, p. 35), and the quality and reliability of the data becomes the responsibility of the user. The general lack of attention to quality in data-driven science has been noted by several scholars, including Bruno Strasser, who comments that

data are turned into knowledge by bioinformaticians and biostatisticians, most of whom have no first hand experience of producing the experimental data they are analyzing. This has contributed to an exaggerated trust in the quality and comparability of the data and to many irreproducible results (Strasser 2012, p. 86).

Biocurators, on the other hand, do have first hand experience from experimental research as they are usually trained biologists with additional experience and education from computer science or informatics (International Society for Biocuration 2018; Leonelli 2016). The rationale behind manually curated data- and knowledge bases is precisely to provide high-quality information so users will save time and avoid inaccuracies and flaws, and entries usually go through several thorough internal review processes and quality controls (e.g., Kerrien et al. 2012; Chen et al. 2019). Manually curated databases are therefore generally assumed to be of high quality (Cusick et al. 2009; Howe et al. 2008).

Transforming insights from scientific datasets and publications into computer-readable formats is far from a matter of simply punching in text and numbers; data and information must be translated into standardized vocabularies and formats. These standardized vocabularies affect the way biological knowledge is represented, and therefore also how biological entities and their interactions are conceived and defined by scientists (Boem 2016), and as biocurators take part in developing the standards and decide how to represent the data, Ankeny and Leonelli argue that biocuration actively influences interpretation and constitutes part of knowledge-creation and production in its own right (Ankeny and Leonelli 2015). Thus, in addition to functioning as reviewers and managers of information, biocurators are also scientific knowledge workers, actively engaging with the data and knowledge they are making accessible to the wider scientific community.

## Precarious trust

In “Trust in Science”, Barber comments that factors like complexity, specialization and the problem of effective surveillance of performance places more emphasis on trust (Barber 1987). As biocurators are highly specialized and perform complex tasks that are not directly visible to the users, trust therefore seems to be crucial. Although Leonelli notes that most users of databases are happy to trust the decisions of biocurators (Leonelli 2016), she also states that biological databases are turning into black boxes where important practices and decisions are hidden from the view of the general users (op.cit.). While the biological databases have become indispensable resources for biological research far beyond the model organism communities, the biocurators themselves seem to be disappearing from the general view and as Burge et al. comment, “how databases are maintained, and by whom, is rather obscure” (Burge et al. 2012, p. 1).

If trust is understood as positive expectations towards the actions and intentions of someone else, the issue of trust in biocurators becomes questionable. According to Luhmann, one has to distinguish between trust and confidence, the latter being the taken-for-granted assumptions that something will work as expected. Trust, on the other hand, requires a previous engagement and presupposes a situation of risk (Luhmann 2000). If users of biological databases are unaware of biocurators and their work and take the quality of available data for granted, which they currently seem to do (Bateman 2010; Baxevanis and Bateman 2015), this is not really a matter of trust in biocurators, but rather a form of confidence in absence of actual knowledge of the system and its participants. Furthermore, it is an absence of awareness of the risks concerning available data, as not all databases provide the same quality. Opening the black box of biological databases in terms of making biocurators and curatorial processes visible is therefore undoubtedly important, and according to the “trusted repositories”-approach of FAIR and Open Science, researchers should be able to evaluate a database, including the trustworthiness of the associated biocurators, in order to decide which resource to choose. However, this requires users to actively look up and assess the information without necessarily having the competence to evaluate the quality of complex curatorial expertise and practices. This is further complicated by the fact that what is considered high-quality data differs between user groups (Wang and Strong 1996; Marchionini et al 2012) and according to a study by Huang et al. (2015), what determines “quality” in data curation also often differs between data users and data curators. Transparency without the proper recognition and support from the scientific community, might therefore undermine the potential trustworthiness of biocurators.

Several scholars have pointed out how trust within science depends on the existence of enduring communities with shared norms and values (Rolin 2002; Edwards 2010), and Olga Kennard, co-founder of the Cambridge Structural Database in 1965, have stated that database organizers had to be well-recognized in the community in order to gain trust (Kennard in Strasser 2011). Biocuration originated in the tight-knit model organism communities where the databases themselves became important mechanisms for fostering of collective trust, both within and between model organism communities (Leonelli and Ankeny 2012).

The model organism communities, including the database curators, fit the definition of *epistemic communities*. According to Peter M. Haas, “[a]n epistemic community is a network of professionals with recognized expertise and competence in a particular domain and an authoritative claim to policy-relevant knowledge within that domain or issue-area” (Haas 1992, p. 3). These communities may consist of members from different disciplines and backgrounds where the members share certain beliefs, values, and notions of validity pertaining to the knowledge and practices in question, as well as a “common policy enterprise-that is, a set of common practices associated with a set of problems to which their professional competence is directed” (ibid.).

As the model organism database curators often had background as laboratory scientists from the community, they were viewed as community members, sharing its interests and values (Leonelli and Ankeny 2012). Unlike the first database curators, however, professional biocurators of today do not necessarily belong to clearly defined epistemic communities. As the life sciences are becoming increasingly interdisciplinary, “communities” are globally distributed and even resources that focus on specific molecules, processes or organisms serve a variety of different epistemic cultures and communities (Leonelli 2016; Oliver et al 2016). According to Leonelli and Ankeny, this allowed the community database curators to become the authorities, reflecting

a form of ceding of responsibility for these types of activities away from individual researchers or particular laboratories, and even away from the communities as previously conceptualized as informally-organized entities, to the databases as the recognized, formal levels of organisation which promote key community functions (Leonelli and Ankeny 2012, p. 34).

However, this authority is not necessarily recognized and acknowledged beyond the database itself. With regards to competence, biocurator expertise is currently not acknowledged by the scientific community. Unlike the closely related field of bioinformatics, there is no formalized degree programs for biocuration (Sanderson 2011) and biocurators tend to be classified as technical staff or service workers (Ankeny and Leonelli 2015).

As Barber notes, assumed competence is not enough to be perceived as trustworthy; it also requires fulfillment of normative obligations to colleagues, institution and the community (Barber 1987). Thus, when curation work is detached from research communities, the loyalties of biocurators could also come under scrutiny. Biocurators operate in arenas that may seem removed from actual science, and in *Data-centric biology* (2016) Leonelli gives an example of how biologists displayed mistrust for the work of biocurators when informed of their practices, complaining that they were biased towards the needs and wishes of computer scientists and not biologists.

## Beyond transparency: reconfiguring trust in data-intensive science

As mentioned in the introduction, Leonelli argues that data-intensive science requires *more* trust, not less, due to the complexity of the data dissemination systems (Leonelli 2016). Although transparency and metadata might limit the need for trust in data producers, trust in those who curate data seems to be unavoidable for data-intensive science as users need to believe that data curators are making good decisions on their behalf in order to be able to rely on data- and knowledge bases. The question should therefore not be how to diminish the need for trust in data-intensive science, but rather how to *facilitate* trust in those who take care of, i.e., curate, data after its production.

In her account of trust in science, philosopher Kristina Rolin claims that a trustworthy moral and epistemic character “is a community achievement and not merely an individual achievement” (Rolin 2002, p. 101) and argues that scientific trust involves “trust in the community’s ability to facilitate inclusive and responsive dialogue based on shared standards of argumentation” (Rolin 2002, p. 100). Community dialogue thus seems to be the key condition for facilitating and maintaining trust, but as noted earlier, biocurators are no longer connected to specific epistemic communities with clearly defined boundaries. As Leonelli and Ankeny (2012) note, cross-species research is now the norm, and the identity politics previously characterizing model organism communities is being challenged as anyone interested can access the databases.

On the other hand, the professionalization of the curator role has opened up for the emergence of new identity: the biocurator, who is not necessarily associated with a specific research community, but with a database or even with a biocuration community. A possible solution could therefore be to view the biocurator community as an epistemic community in its own right, i.e., as a “a network of professionals with recognized expertise and competence in a particular domain and an authoritative claim to policy-relevant knowledge within that domain or issue-area” (Haas 1992, p. 3). It would then be the responsibility of the biocuration community to maintain formal and informal trust relations and to facilitate dialogue both within the community itself as well as with different user groups. There are already steps being taken in this direction; biocurators have been meeting regularly since 2003 (Harding 2006), and in 2009, the *International Society for Biocuration* (ISB) was founded in order to provide a forum for networking and to promote increased awareness of biocuration and biological databases (Bateman 2010). Through various forms of outreach, the ISB encourages user communities to collaborate with biocurators, for instance through *community curation*, where researchers do the initial curation and professional biocurators takes care of quality control (International Society for Biocuration 2018).

As life science research depends on high-quality biological databases, the construction of an epistemic community capable of ensuring the trustworthiness of its members should not be an issue left solely to biocurators but receive support and recognition from the greater scientific community which they serve. Currently, biological databases and biocurators are facing challenges regarding the general lack of recognition and funding allocated to curation activities. The maintenance of existing data and information is seldom considered as important as the generation of new, and funding for both model organism databases and other types of databases is in constant danger of being reduced (Chen et al. 2019). By recognizing biocuration as an important and necessary part of the scientific process and by ensuring proper funding and representation, policy makers, funders and the greater scientific community could therefore facilitate the reconfiguration of existing scientific trust mechanisms and support biocurators as trustworthy scientific actors.

## Concluding remarks

As science is becoming more data-intensive and collaborative, the issue of trust is becoming more challenging than ever. With the increasing importance of databases and the detachment of data and information from its original context, the scientific trust chain should also include those who take care of data after its production, but in current science policies, the importance of trust is diminished in favor of openness and transparency. Data curation is seldom mentioned, and when it is, it seems to be considered a mainly technical task. Added-value curation like biocuration, however, involves enriching and transforming original data and literature, and requires considerable scientific expertise. Openness and transparency are therefore not in any way making trust redundant. Quite the contrary; as the levels of knowledge, judgement and skills necessary for scientific data curation are revealed, the need for robust relations of trust within data-intensive science seems to be more pertinent than ever.

With its deep and complex level of curation, biocuration might not be representative for the majority of the vast body of digital data curation practices currently taking place in different scientific domains. However, as the life sciences are often used as an example of successful and promising data-intensive infrastructures, they could serve as an example of how trust needs to be reinforced in order to make data-intensive science succeed across disciplinary borders. At the moment, neither current nor envisioned life sciences are equipped with mechanisms to secure and maintain this trust, but as one important function of trust is to reduce transaction costs, the investments in facilitating trust in databases and digital curators would be easily returned by the time and effort saved on having immediate access to high-quality data.

Trust in science should therefore not be diminished, but rather rethought and reconfigured. Policymakers as well as funding agencies and the greater scientific community should award attention and authority to digital curator communities, and mechanisms to establish and maintain trust in digital data curators should be embedded in the envisioned infrastructures for data-intensive science. Such a reconfiguration of trust will, however, require a more nuanced understanding of how data-intensive science works and of the importance of digital curation than currently seen in the strategies and policies promoting open data-intensive science.
